# Randomized feature selection based semi-supervised latent Dirichlet allocation for microbiome analysis

**DOI:** 10.1038/s41598-024-59682-4

**Published:** 2024-04-17

**Authors:** Namitha Pais, Nalini Ravishanker, Sanguthevar Rajasekaran, George Weinstock, Dong-Binh Tran

**Affiliations:** 1 Department of Statistics, University of Connecticut, Storrs, CT USA; 2 Department of Computer Science and Engineering, University of Connecticut, Storrs, CT USA; 3 Jackson Laboratory for Genomic Medicine, Farmington, CT USA

**Keywords:** Diseases, Medical research, Mathematics and computing

## Abstract

Health and disease are fundamentally influenced by microbial communities and their genes (the microbiome). An in-depth analysis of microbiome structure that enables the classification of individuals based on their health can be crucial in enhancing diagnostics and treatment strategies to improve the overall well-being of an individual. In this paper, we present a novel semi-supervised methodology known as Randomized Feature Selection based Latent Dirichlet Allocation (RFSLDA) to study the impact of the gut microbiome on a subject’s health status. Since the data in our study consists of fuzzy health labels, which are self-reported, traditional supervised learning approaches may not be suitable. As a first step, based on the similarity between documents in text analysis and gut-microbiome data, we employ Latent Dirichlet Allocation (LDA), a topic modeling approach which uses microbiome counts as features to group subjects into relatively homogeneous clusters, without invoking any knowledge of observed health status (labels) of subjects. We then leverage information from the observed health status of subjects to associate these clusters with the most similar health status making it a semi-supervised approach. Finally, a feature selection technique is incorporated into the model to improve the overall classification performance. The proposed method provides a semi-supervised topic modelling approach that can help handle the high dimensionality of the microbiome data in association studies. Our experiments reveal that our semi-supervised classification algorithm is effective and efficient in terms of high classification accuracy compared to popular supervised learning approaches like SVM and multinomial logistic model. The RFSLDA framework is attractive because it (i) enhances clustering accuracy by identifying key bacteria types as indicators of health status, (ii) identifies key bacteria types *within each group* based on estimates of the proportion of bacteria types within the groups, and (iii) computes a measure of within-group similarity to identify highly similar subjects in terms of their health status.

## Introduction

Humans coexist with trillions of single-cell organisms living within their bodies, labeled as human microbiota or microbiome. Most of these organisms reside in the human gut, where most are bacteria, although viruses and fungi are also part of the microbiome. These microbes can be symbiotic or pathogenic and coexist without conflict in a healthy body. However, changes in diet, antibiotics, etc., can disturb this balance, making the body more susceptible to diseases. Recent advances in human microbiome research show evidence of the impact of microbiomes on the host’s well-being^1,2^. The objectives of the research are to characterize the composition of a normal microbiome in healthy individuals, and to investigate similarities and differences between individuals by characterizing microbiomes based on their core functions, ecological characteristics, or temporal dynamics.

With recent developments in machine learning techniques, researchers have adopted several computational approaches to diagnose and understand the microbiome data and its implications on human health. Topçuoğlu et al.^3^ discussed modeling techniques for microbiome profiling to obtain a biologically-interpretable mathematical formula for predicting the likelihood of disease. Marcos-Zambrano and Gupta et al.^4,5^ discussed and compared standard machine learning approaches in terms of predictive accuracy and interpretability. Significant advances in microbiome research are being made in order to address characteristics of the human microbiome structure such as high diversity and presence of rare bacteria types, as well as to handle insufficient samples of individuals at risk of several diseases^6,7^. Several applications of machine learning techniques in microbiome studies also incorporate feature selection algorithms to identify bacteria types associated with various diverse health conditions. For instance, Lee et al.^8^explores a machine learning-based recursive feature elimination (RFE) approach to identify strong biomarkers for inflammatory bowel disease (IBD). Similarly, Chen et al.^9^ discusses a novel feature selection method in combination with machine learning methods to detect patterns and predict conditions related to oral microbiota in periodontal disease. Leske et al.^10^ presents a bi-objective genetic algorithm known as BiGAMi designed for feature selection in microbial datasets, that trains high-performing phenotype classifiers. In addition to the machine learning techniques, mixed membership models such as Latent Dirichlet Allocation (LDA) have also been applied to the microbiome studies to identify latent subcommunities of microbial species. In particular, Deek et al.^11^ discusses a zero-inflated Latent Dirichlet Allocation Model (zinLDA) applied to American Gut Project data to identify microbial communities characterized by different bacterial genera. The logistic-tree normal (LTN) model incorporated into LDA in LeBlanc and Ma^12^ discusses an extension of LDA to identify microbial subcommunities by incorporating cross-sample heterogeneity or random effects in the model. While these methods aim to identify the sub-communities, the association of these sub-communities with health status has not been extensively studied. To address this, we develop a feature selection-based topic modeling approach (semi-supervised) that is in the nature of feature selection-based machine learning methods (which are supervised).

Our paper discusses a novel semi-supervised topic modeling approach that analyzes patterns in the microbiome data to identify relatively homogeneous groups and compares their association with observed health status (fuzzy labels), to classify subjects based on health status. We begin by exploring an unsupervised topic modeling approach that provides a powerful tool for discovering and exploiting the hidden structure in the microbiome data. Given the microbiome counts in the subject’s gut, we are interested in checking whether these counts can be used as features to group (cluster) subjects without any information about their health status. Subsequently, we can classify the subjects into different health status levels by assessing the similarity between the observed health status and the clusters. We develop a methodology called “Randomized Feature Selection based Latent Dirichlet Allocation” (RFSLDA) that identifies important bacteria types to distinguish between different levels of health status and classify subjects based on their counts. Our method (i) provides a semi-supervised topic modeling approach to classify subjects into different health status based on their gut-microbiome composition and, (ii) provides a feature selection technique to identify important bacteria types from the high dimensional microbiome data to improve model performance substantially. Experimental results indicate that our algorithm performs well.

## Data description

We analyze data provided by the Jackson Laboratory in Farmington, CT to examine how microbiomes affect human health. Medical professionals collected blood from $M=89$ subjects containing healthy individuals and individuals with prediabetes for host molecular omics profiling. They also collected two types of samples (stool and nasal swabs). Microbiome profiling then recorded counts on each $B=109$ bacteria profiled using 16S gene sequencing. The detailed description of the raw data is provided in^13^. Suppose $Y_{i,\ell }$ for $i=1,2,\ldots ,M$, and $\ell = 1,2,\ldots , B$ represents a count of the $\ell$th type of bacterium of the *i*th subject. Initial exploration shows that the observed read counts $Y_{i,\ell }$ exhibit a wide range of values. Following expert opinion that it may be counter-productive to include all bacterial types into the data analysis, we identify *top* bacterial types based on two measures, i.e., *abundance* and *prevalence*. While abundance captures the composition of bacteria types in the microbiome, prevalence captures the presence/absence of bacteria types above a given detection threshold.

Let $\mathbb {P}=\{p_{i,\ell }\}$ be an ${M \times B}$ matrix of proportions of the $\ell ^{th}$ bacterium in subject *i*, where,

1 $$\begin{aligned} p_{i,\ell }=\dfrac{Y_{i\ell }}{\sum _{\ell =1}^{B}Y_{i\ell }}. \end{aligned}$$

For bacterial type $\ell = 1,2,\ldots , B$, abundance and prevalence are defined by

2 $$\begin{aligned} \mathscr {A}_\ell&=\sum _{i=1}^M p_{i,\ell }, \text { and } \end{aligned}$$

3 $$\begin{aligned} \mathscr {P}_\ell&=\sum _{i=1}^M I_{i\ell }, \text { where } I_{i,\ell }=\textbf{1}[p_{i,\ell } \ge \omega ]. \end{aligned}$$

Supplementary Figs. S1 and S2 shows the abundance and prevalence for the $B=109$ bacteria types respectively. We see that some types are highly abundant, while some (possibly other) types are highly prevalent.

**Figure 1:**
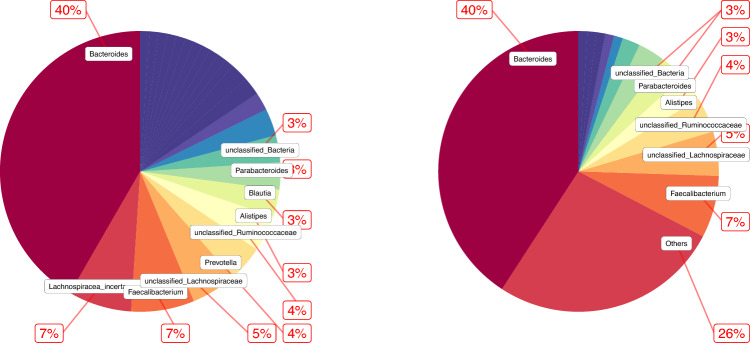
Pie charts represent total percentages of average read counts taken over all the subjects for (i) all the 109 bacteria types and (ii) the top 50 bacteria types obtained using the Tau Path Method.

We use the tau-path method^14,15^ for identifying the top $K=50$ bacteria types determined by the high concordance between their abundance and prevalence measures (details are shown in the Appendix). Discussion with medical experts confirmed that no significant biological features were filtered out using the tau-path method. We aggregate the remaining bacteria types into a single category labeled “Others”. Let $B_0= K+1=51$. Let $Z_{i,l}$, $i=1,2,\ldots ,M$ and $\ell =1,2,\ldots ,B_0$ denote counts on the top bacteria types on *M* subjects. The left plot in Fig. 1 displays the distribution of all bacteria based on the average read counts across all subjects, while the right plot shows that the the top bacteria types have reasonably sufficient read counts.

Alternatively, another way to pre-process the data involves independently applying the tau-path method to each class-specific data set based on health status and selecting the top $K=50$ features for each subset. These features can then be combined and used for further analysis. The remainder of the paper focuses on the results obtained from the tau-path pre-processing step, which identifies top-*K* features that are globally prevalent and abundant. The results of the alternative pre-processing method are available from https://github.com/NamithaVionaPais/RFSLDA.

In the following sections, we provide details on our RFSLDA algorithm which uses microbiome read counts on $B_0 = 51$ types to group subjects based on their health status. For convenience, Supplementary Table S1 presents the notations used in the rest of the article.

## Randomized feature selection based latent Dirichlet allocation

This section describes our algorithm for classifying subjects into different groups which characterize the relationship between their gut micriobiome and health status. First, we build a latent Dirichlet allocation (LDA) model which uses only the microbiome counts as features to determine relatively homogeneous clusters in an *unsupervised way*, yielding latent topic labels for the subjects (Sect. 3.1.) Second, we do *semi-supervised LDA* to match information on observed health-status labels of subjects with their latent topic labels from Sect. 3.1 in order to classify them into different levels (see Sect. 3.2). A final *feature selection* step in Sect. 3.3 helps us to identify bacteria types which optimally drive the classification of subjects into suitable health status groups. We conduct the analysis in R and the code can be accessed from https://github.com/NamithaVionaPais/RFSLDA.

### Unsupervised latent Dirichlet allocation

Latent Dirichlet Allocation (LDA)^16^ is an unsupervised, mixed-membership model mainly used in document analysis. LDA assumes *T* unobserved topics (clusters) associated with a collection of subjects where each subject exhibits these topics in different proportions. This model uses the observed microbiome counts as features to infer the hidden topic structure of each subject. Suppose we have a corpus $\mathscr {C}$ consisting of *M* subjects where the observed microbiome counts on each of the subjects are represented as $\mathscr {D}={(b_{1},b_2,\ldots ,b_{B_0})}$. If $N=\sum _{l=1}^{B_0}b_{l}$ is the total microbiome count, then one can also represent $\mathscr {D}$ as $\mathscr {D}={(\varvec{w}_{1},\varvec{w}_2,\ldots ,\varvec{w}_N)}$ where $\varvec{w}_n$ corresponds to the $n^{th}$ bacterium present in the subject’s gut represented by a $B_0 \times 1$ vector corresponding to the $v^{th}$ bacteria type such that $w^v=1$ and $w^u=0$ for $u\ne v$. LDA assumes the following generative process for each subject $\mathscr {D}_d$ with total microbiome count $N_d$, for $d \in \{1,2,\ldots M\}$ present in the corpus $\mathscr {C}$:

1. Choose $$N_d \sim Pois(\lambda )$$
2. Choose $\varvec{\theta }_d\mid \varvec{\alpha } \sim Dir(\varvec{\alpha })$ where, $\varvec{\theta }_d=(\theta _1,\theta _2,\ldots ,\theta _J)$ and *Dir*(.) is a symmetric Dirichlet distribution.
3. For each of the $N_d$ bacterium, $\varvec{w}_{d,n}$
  - Choose a topic $$\varvec{Z_{d,n}} \mid \varvec{\theta }_d \sim Mult(1,\varvec{\theta _d})$$
  - Choose a bacterium ${\varvec{w}_{d,n} }\mid \{\varvec{Z}_{d,n},\varvec{\beta }\}\sim Mult(1,\varvec{\beta }_{\varvec{Z}_{d,n}})$, a multinomial probability distribution conditioned on the topic $\varvec{Z}_{d,n}$.

We use the LDA model to obtain $T=3$ hidden topics (clusters) using the read counts from the $B_0=51$ bacteria types on $M=89$ subjects. We choose $T=3$ clusters in order to associate them with the observed health status with $C=3$ levels. One can explore different values of *T* by re-running the model with different numbers of topics ranging from, say, 2 to 20, and then choose the optimal number of topics *T* that yields the best fit for the data based on criteria called perplexity defined in^16^. Once the structure of the model is defined, the goal is to estimate the model parameters and compute the posterior distribution for inference. The LDA model estimation is done using the R package *topicmodels*^17^, which uses variational expectation maximization (VEM) algorithm to estimate the model parameters and variational inference (VI) algorithm to approximate the posterior distribution. Details are shown in the Appendix.

The latent topical structure is represented by the estimated topic proportions $\pi ^{'}_{t,i}$ on each subject, for $i= 1,2,\ldots , M$ and $t=1,2,\ldots T$. Estimating the per-subject topic proportion using LDA allows us to associate subjects with multiple topics, unlike many clustering algorithms that assign one topic per subject. In addition, the topic with the highest proportion is assigned as the topic label $\pi _i=argmax\{\pi ^{'}_{t,i}\}$ for a given subject. While $\pi _i$ enables us to group the subjects into *T* topics (clusters), $\pi ^{'}_{t,i}$ helps us compare the degree of similarity of subjects within clusters.

Using the LDA model, we group 57 subjects into cluster 1, 15 subjects into cluster 2 and 17 subjects into cluster 3 (These clusters via their compositional proportions $\pi ^{'}_{t,i}$ are shown in Supplementary Fig. S3).

### Semi-supervised latent Dirichlet allocation

In addition to the bacteria counts, we obtain information on observed health status $C_{i}$ for each subject. These were obtained by the medical professional through a battery of molecular and clinical laboratory tests, complemented by self-reported online surveys which documented changes in medication, physical activity, diet preference, and perceived stress level. Being self-reported, the health status levels can be considered *fuzzy* labels that provide only ballpark information about an subjects’s health. We have retained the health status levels *Healthy* and *Infection*, but grouped a few levels with insufficient data that indicated medical stress (immunization, antibiotics, travel, fiber, colonoscopy, surgery, weight gain, weight loss, stress, and allergies) into a single level, *Stress*. Despite the rare occurrence of the level *infection*, (Supplementary Fig. S4) it is an extremely important level to identify.

We bring in the information from the observed health labels to provide a qualitative interpretation of the clusters learned from the topic model approach. We predict the health status levels of subjects by associating the *topic labels* $\pi _i$ to the *observed* health status ${C_i}$, for $i= 1,2,\ldots , M$. This is achieved by considering all the *T*! matches between the topic labels and the observed health status (Supplementary Fig. S5 shows three possible matches).The choice to consider all possible T! matches is made to prevent any bias resulting as we do not have any prior information on associating topic to health status. However, in the event we have any a priori knowledge, such as knowing that the sub-communities of bacteria types in topic 1 can only be present in subjects with health status with infection/stress, we can incorporate this information to reduce the number of possible matches from T! = 6 to just four possible combinations.

For each of the *T*! scenarios we calculate a classification metric defined as,

4 $$\begin{aligned} A_w= \sum _{t=1}^T w_t\times {TPR_t}, \end{aligned}$$

where $w_1,w_2,w_3$ are weights such that $\sum _{i=1}^T w_t=1$, and $TPR_t$ corresponds to the true positive rate (TPR) indicating the proportion of correct predictions for class *t* where, $t=1,2,\ldots , T$. Since we have unbalanced classes (see Supplementary Fig. S4), we use weighted accuracy $A_w$ as a metric. The subjects are then classified into different health status levels based on the optimal match identified by the metric. As a result, our framework becomes semi-supervised.

We conduct a grid search (Supplementary Table S2) and set $w_1=0.6, w_2=0.15$ and $w_3=0.25.$ Considering the subject-specific microbiome data with $B_0=51$ bacterial types, we are able to only achieve a weighted accuracy of $46.67\%$ with just one infected subject correctly classified. The corresponding confusion matrix is shown in Table 1a.

### RFSLDA algorithm

We incorporate a feature selection technique^18^ into the semi-supervised LDA model in Sect. 3.2 in order to identify an optimal subset of bacteria types and improve model performance. That is, we identify a subset of the $B_0$ bacteria types that are most important in improving the LDA based classification for determining a subject’s health status, by eliminating bacteria types which reduce the predictive power of the classifier. We develop a *Randomized Feature Selection based Latent Dirichlet Allocation*(RFSLDA) algorithm; see Algorithm 1.

**Algorithm 1:**
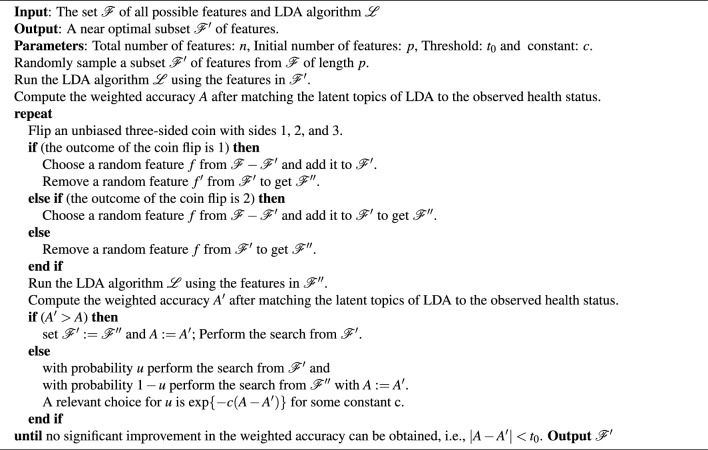
Randomized Feature Selection based Latent Dirichlet Allocation.

We set $n=B_0$ and use grid search to set $c=1$ , $p=\dfrac{n}{2}$ and $t_0=0.0001$ (Supplementary Table S3) to run the RFSLDA algorithm. We repeat Algorithm 1 $R=50$ times to ensure that the optimal subset of bacteria types chosen is not sensitive to the choice of the initial subset. At each iteration, we record optimal set of bacteria types and the corresponding accuracy. We identify the iteration with the highest weighted accuracy and select the corresponding subset of bacteria types as the optimal subset. This step improves the overall weighted accuracy of grouping to $72.12\%$, which is significantly higher than the weighted accuracy of $46.67\%$ (shown in Sect. 3.2) we obtained before feature selection. The corresponding confusion matrix is shown in Table 1b.

**Table 1:** Confusion matrices obtained using data from $M=89$ subjects.

	H	I	S
(a) Semi-supervised LDA			
H	44	4	9
I	10	1	4
S	12	1	4
(b) RFSLDA			
H	50	1	5
I	8	4	3
S	8	1	9

In the RFSLDA algorithm we start with a random subset $\mathscr {F}^{'}$ of the features from the feature space and calculate the weighted accuracy *A* from the semi-supervised LDA model corresponding to this subset. Next, we sample a value randomly from the set $\{1, 2, 3\}$, with each number having an equal probability of being chosen. This can be considered as flipping an unbiased three sided coin with sides 1, 2, and 3. If the outcome of the coin flip is 1, we choose the random subset $\mathscr {F}^{''}$ by removing one feature $\mathscr {F}$ from and adding a new feature from $\mathscr {F}-\mathscr {F}^{'}$. After choosing $\mathscr {F}^{''}$, we compute its weighted accuracy $A^{'}$ from the semi-supervised LDA model. If $A^{'}>A$, we move to the random subset $\mathscr {F}^{''}$ and proceed with the search from $\mathscr {F}^{''}$. On the other hand, if $A^{'}\le A$, then we stay with the random subset $\mathscr {F}^{'}$ (with some probability *u*) or move to the random subset $\mathscr {F}^{''}$ with probability $(1-u)$. This step is done to ensure that we do not get stuck in a local maximum. If the outcome of the coin flip is 2, we choose a random subset $\mathscr {F}^{''}$ by removing one feature from random subset $\mathscr {F}^{'}$ and compute its accuracy its weighted accuracy $A^{'}$. In the case where the outcome of the coin flip is 3, we choose a random subset $\mathscr {F}^{''}$ by adding one feature to $\mathscr {F}^{'}$ and computing its weighted accuracy $A^{'}$. The next steps for case 2 and 3 are the same as stated in the case of 1. This process of searching the space is continued until no significant improvement in the accuracy can be obtained. The RFSLDA algorithm correctly classifies four out of six infected subjects (which is a crucial in medical practice) and improves the overall model performance. It may be less problematic that a few healthy subjects are misclassified as infected or stressed.

## Interpreting results from the RFSLDA algorithm

We present the interpretation of results from our RFSLDA algorithm.

### Optimal bacteria types by health status levels

**Figure 2:**
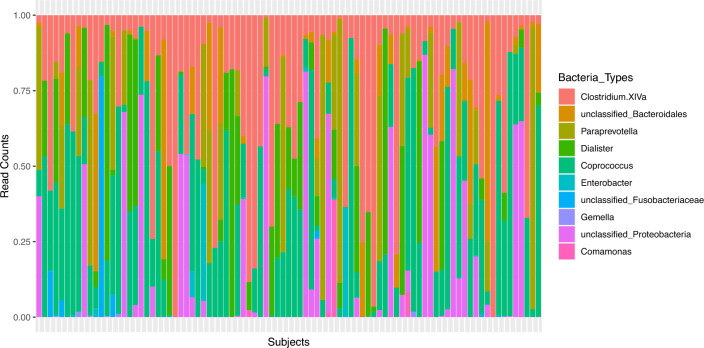
Relative abundance of optimal bacteria types across subjects.

The feature selection step of the RFSLDA algorithm enables us to select a subset of the bacteria types that are the most important in determining the health status. These are: *Clostridium.XlVa, unclassified_Bacteroidales, Paraprevotella, Dialister, Coprococcus, Enterobacter, unclassified_Fusobacteriaceae, Gemella, unclassified_Proteobacteria and, Comamonas*. The composition of these bacteria types on each of the subjects are shown in Fig. 2. This figure shows the relative abundance of optimal bacterial types across all subjects. We compare the optimal Bacteria types obtained from RFSLDA with the important bacteria types identified by a conventional method, such as Boruta using the R package *Boruta*^19^. Among the three important features identified by the Boruta method (*Eggerthella*, *Paraprevotella*, and *unclassified_Proteobacteria*) two of them are present in the optimal set identified by RFSLDA.

**Figure 3:**
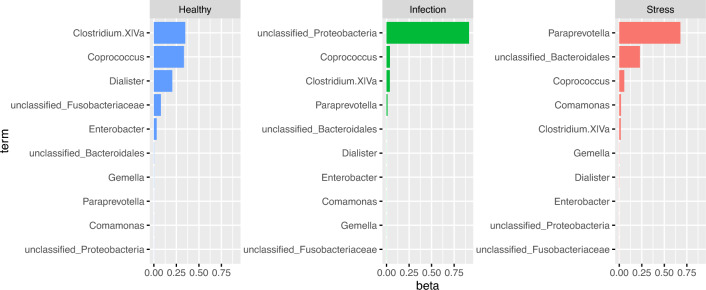
Top bacteria across different health status with their estimated proportions.

From the RFSLDA algorithm, we obtain estimates on the distribution of the different bacteria types by health status levels. Figure 3 shows the selected bacteria types across each health status level, along with their estimated proportions. We see that unclassified_Proteobacteria is most commonly found in subjects classified under infection, with an estimated proportion of 0.9, while Paraprevotella is more commonly found in subjects classified as stress, with an estimated proportion of 0.67. Among healthy subjects, the bacteria types Clostridium.XlVa and Coprococcus are predominant, with estimated proportions 0.35 and 0.33, respectively.

The results from Section 4.1 as an exploratory analysis to plan experiments which test and confirm if the selected bacteria types have a causal effect or a random correlation on health status. This analysis serves useful to narrow down the bacteria types to be investigated.

###  Within group similarity of subjects by health status

We evaluate the within-class similarity using information from the estimated topic structure $\pi ^{'}_{t,i}$. Let $\pi _{i,[1]}$ and $\pi _{i,[2]}$ denote the highest and second highest topic proportions for each subject and let $\lambda =0.7$ denote a user-specified threshold.

Suppose we consider the subjects classified as healthy.

1. Subjects with maximum estimated topic proportion $\pi _{i,[1]} \ge \lambda$ are grouped as similar, and called *Predominantly Healthy*.
2. Subjects with $\pi _{i,[1]}< \lambda$, and $\pi _{i,[2]}$ associated to health status level *infection* (or,*stress*) are grouped similar and called *Healthy-Infection* (or, *Healthy-Stress*).

As an example, suppose we have four subjects with estimated topic proportions associated to stress, healthy, and infection as $S_1 = (0.2,0.75,0.05)$, $S_2 = (0.15,0.5,0.35)$, $S_3 = (0.001,0.9,0.09)$, and $S_4 = (0.2,0.4,0.4)$. Each subject is classified as healthy according to the maximum estimated topic proportion. However, within the healthy group, we are able to identify that Subjects 1 and 3 are more similar to each other, and Subjects 2 and 4 are more similar to each other.

**Figure 4:**
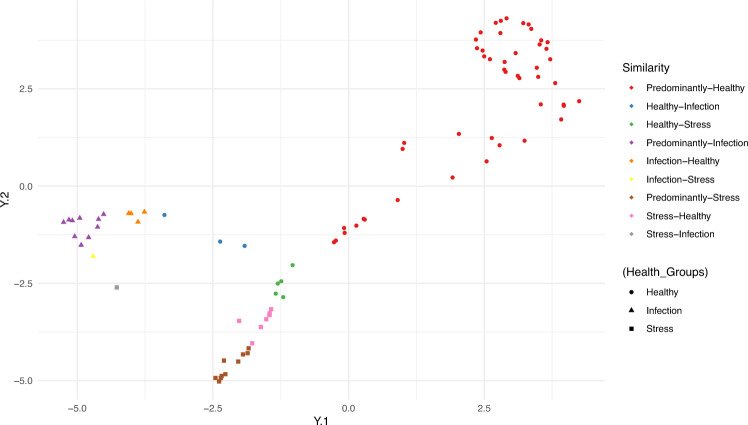
Visualizing the within-group similarity across each health status level using the t-SNE method.

To visualize three-dimensional topic proportions, we employ the t-distributed stochastic neighbor embedding (t-SNE) method^20^, to represent similar objects by nearby points and dissimilar objects by distant points. The t-SNE plot is shown in Fig. 4. We can use this plot to identify subjects within each health group that are more similar to each other.

## Comparison to other supervised learning methods

The semi-supervised RFSLDA method outperforms popular supervised learning methods like the multinomial logistic model^21^, support vector machines (SVM)^22^, XGBoost^23^ and Neural Network (NN). To illustrate this, we run all three methods using an 80-20 train-test split of the data using the same set of features selected in the RFSLDA method. We conduct this analysis within a k-fold cross-validation framework using k=5 folds. It is to be noted that while implementing RFSLDA, the matching of the health status to the topics are done as a part of the training process. These matches are then used as associated topic class labels directly on the test data. We fit the five methods to the training data with $M=69$ subjects, and evaluate the fits on the test data with $M=20$ subjects. The multinomial logit model is implemented using the R package *nnet*^24^. The SVM (with a linear kernel) is implemented using the R package *e1071*^25^. The XGBoost is implemented using R package *xgboost*^23^. The NN (with three hidden layers) is implemented using R package *neuralnet*^26^.

**Table 2:** $5-$fold evaluation metrics comparing the multinomial logit, SVM, NN , XGboost and RFSLDA on the train and test framework.

	Multinomial-logit	SVM	NN	XGBoost	RFSLDA
Weighted precision (train)	0.6663	**0.7385**	0.6701	0.4882	0.6559
Weighted precision (test)	0.5339	0.5463	0.4599	0.4386	**0.6629**
Weighted accuracy (train)	0.6573	**0.7436**	0.7078	0.6131	0.6577
Weighted accuracy (test)	0.5574	0.5994	0.5202	0.5815	**0.6804**
Weighted recall (train)	0.6556	**0.7728**	0.7032	0.6125	0.6547
Weighted recall (test)	0.5564	0.5977	0.5198	0.5815	**0.6774**

E.g. Significant values are in [bold]

Table 2 shows the average weighted accuracy, average weighted precision and average weighted recall values for the train and test data sets evaluated using k fold cross validation (with five folds). The SVM performs better than the other methods on the train data. However the usefulness of an approach and generalizability is best assessed by its performance on a test data. From Table 2 we observe that RFSLDA performs substantially better than the supervised methods on the test data.

## Optimal bacteria types after data balancing

**Figure 5:**
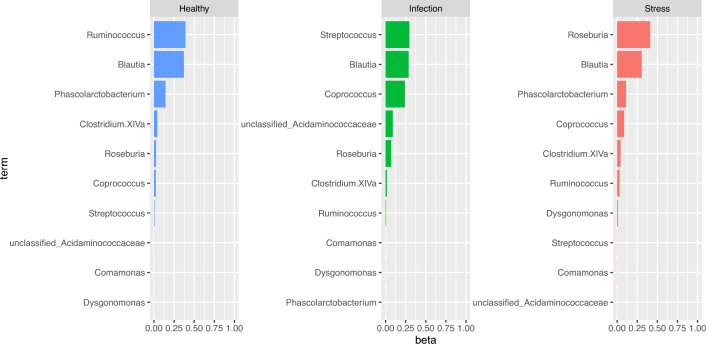
Top bacteria across different health status with their estimated proportions for the balanced data.

The gut-microbiome dataset used in this study is imbalanced in the response variable, health status, as shown in Fig. 2. Therefore, in order to identify optimal bacteria types under a balanced framework, we implement data balancing technique on the original dataset to obtain a data which is balanced across the levels of the health status. The original dataset contains $M=89$ subjects with $K=51$ microbiome counts. The data balancing technique results in a balanced dataset comprising $M=90$ subjects and maintaining $K=51$ microbiome counts, where the distribution across different health statuses is now even (30 observations in each level). The data balancing procedure is done using the Synthetic Minority Over-sampling Technique (SMOTE) and Cluster-based Undersampling (SCUT) hybrid sampling technique^27^. The SCUT hybrid sampling technique oversamples minority classes by creating synthetic examples for the minority class. The synthetic data is generated by (i) calculating the difference between a minority sample and its nearest neighbor, and (ii) scaling the difference and adding to the minority sample to enlarge the decision region for the minority class. For under sampling the majority class, the SCUT hybrid sampling technique employs cluster analysis to identify sub-clusters, to ensure at least one instance is selected from each sub-cluster while undersampling. This ensures that the undersampling provides a better representation of the original data. We implement the SCUT technique using the R package *scutr*^27^. We run the RFSLDA algorithm (Algorithm 1) with accuracy as the metric of comparison to obtain the optimal bacteria types selected by the RFSLDA method on the balanced data. The optimal bacteria types selected by RFSLDA include *Blautia, Coprococcus, unclassified_Acidaminococcaceae, Streptococcus, Dysgonomonas, Ruminococcus, Comamonas, Roseburia, Phascolarctobacterium, Clostridium.XlVa*. Figure 5 shows the selected bacteria types across each health status level, along with their estimated proportions. We see that Ruminococcus is most commonly found in subjects classified as healthy, with an estimated proportion of 0.4, Streptococcus is more commonly found in subjects with infection with an estimated proportion of 0.3. Roseburia is commonly found in subjects with health status stress with an estimated proportion of 0.4.

We evaluate the performance of RFSLDA in comparison to other supervised learning methods, using an 80-20 train-test split of the balanced data with the same set of features selected in the RFSLDA method in a k-fold cross validation framework. Table 3 shows the average accuracy, average precision and average recall values for the train and test data sets evaluated using k fold cross validation (with five folds). While the SVM performs better than the other methods on the train data and logit model performs better than all methods in the test data, RFSLDA performs reasonably well in the test data in comparison to NN, XGBosst and SVM.

**Table 3:** $5-$fold evaluation metrics comparing the multinomial logit, SVM, NN , XGboost and RFSLDA on the train and test framework on the balanced data.

	Logit	SVM	NN	XGBoost	RFSLDA
Precision (train)	0.8078	**1.0000**	0.5952	0.8639	0.6529
Precision (test)	**0.7423**	0.6573	0.4258	0.6288	0.6857
Accuracy(train)	0.8083	**1.0000**	0.4917	0.8528	0.6444
Accuracy(test)	**0.7222**	0.4222	0.4444	0.6111	0.6444
Recall (train)	0.8083	**1.0000**	0.4917	0.8528	0.6444
Recall (test)	**0.7222**	0.4222	0.4444	0.6111	0.6444

E.g. Significant values are in [bold]

## Discussion and conclusions

The current study using subject-specific data reveals that gut microbes play a fundamental role in human health. As an initial data preprocessing, we determine the top bacteria levels from the zero-inflated microbiome data based on abundance and prevalence to filter out potentially uninformative bacteria types using the tau-path method. The RFSLDA method then uses a semi-supervised LDA model to classify subjects based on the health status. Through a feature selection incorporated in our framework, we identify significant bacteria types that can accurately distinguish between different health statuses and enhance the accuracy of classification. We identify the top bacteria types in each class. Unclassified_Proteobacteria, for instance, is predominant in subjects classified as infected, but rare in subjects classified as healthy and stress. Further, using the estimated topic structure we obtain a within group similarity of subjects by the health status levels. In addition, since the observed data is imbalanced in the levels of the response variable health status, we perform data balancing using SCUT hybrid sampling technique and perform RFSLDA on the balanced data to identify the top bacteria types across each level of the health status.

The results of a comparative study demonstrate that our RFSLDA method, despite being a semi-supervised approach, outperforms traditional supervised methods, such as SVM and Multinomial logit in identifying rare yet crucial health levels (infected, stress) in the test data. This is an important finding. In comparison to extensions of LDA in microbiome studies discussed in^11,12^ that identifies important microbial sub-communities, our method associates the latent microbial sub-communities obtained from LDA with the subject’s health status. Our results enable practitioners to identify the specific types of bacteria associated with each health status, providing valuable insight into the intricate connections between gut microbiome and human health. Under the balanced data, while the multinomial logit outperforms the RFSLDA in the test data, the RFSLDA performs reasonably well on the test data in comparison to neural network, XGBoost and SVM models.

The present study has some limitations. First, despite showing a significant relationship between gut microbiome profiles and health status, our model misclassifies $25\%$ of healthy people as infected or stressed. Although we acknowledge this shortcoming, since the model is able to detect 4 out of 6 infected subjects correctly, we are willing to accommodate these false negatives. Moreover, since fuzzy labels are considered to represent the true observed health status of subjects, it could be possible for them to self-report themselves as healthy when they are not. Thus, it is imperative to investigate these misclassifications more closely in order to gain a deeper understanding of their health condition. Future research can also further investigate the causal relationship between the gut microbiome and human health using a supervised topic modeling approach. Our RFSLDA approach can also be extended to a longitudinal data setup, where subjects visit the facility at different times.

### Supplementary Information

Supplementary Information.

## Data Availability

Raw data included in this study were provided by the Jackson Laboratory in Farmington, CT. The original study is a part of the NIH Human Microbiome 2 project, see https://portal.hmpdacc.org.

## References

[1] Cho Ilseung BMJ (2012). The human microbiome: At the interface of health and disease. Nat. Rev. Genet..

[2] Lloyd-Price Jason HC, Galeb Abu-Ali (2016). The healthy human microbiome. Genome Med..

[3] Topçuoğlu BD (2020). A framework for effective application of machine learning to microbiome-based classification problems. MBio.

[4] Marcos-Zambrano LJ (2021). Applications of machine learning in human microbiome studies: A review on feature selection, biomarker identification, disease prediction and treatment. Front. Microbiol..

[5] Gupta VK (2020). A predictive index for health status using species-level gut microbiome profiling. Nat. Commun..

[6] Pflughoeft KJ, Versalovic J (2012). Human microbiome in health and disease. Annu. Rev. Pathol..

[7] Berg G (2020). Microbiome definition re-visited: Old concepts and new challenges. Microbiome.

[8] Lee Y, Cappellato M, Di Camillo B (2023). Machine learning-based feature selection to search stable microbial biomarkers: Application to inflammatory bowel disease. GigaScience.

[9] Chen W-P (2018). Composition analysis and feature selection of the oral microbiota associated with periodontal disease. BioMed Res. Int..

[10] Leske M, Bottacini F, Afli H, Andrade BG (2022). BiGAMi: Bi-objective genetic algorithm fitness function for feature selection on microbiome datasets. Methods Protoc..

[11] Deek RA, Li H (2021). A zero-inflated latent Dirichlet allocation model for microbiome studies. Front. Genet..

[12] LeBlanc P, Ma L (2023). Microbiome subcommunity learning with logistic-tree normal latent Dirichlet allocation. Biometrics.

[13] Zhou W (2019). Longitudinal multi-omics of host-microbe dynamics in prediabetes. Nature.

[14] Zhang Y, Ravishanker N, Ivan J, Mamun S (2018). An application of the Tau-Path method in highway safety. J. Indian Soc. Probab. Stat..

[15] Yu L, Verducci JS, Blower PE (2011). The Tau-Path test for monotone association in an unspecified subpopulation: Application to chemogenomic data mining. Stat. Methodol..

[16] Blei DM, Ng AY, Jordan MI (2003). Latent Dirichlet allocation. J. Mach. Learn. Res..

[17] Grün B, Hornik K (2011). topicmodels: An R package for fitting topic models. J. Stat. Softw..

[18] Saha S, Rajasekaran S, Ramprasad R (2015). Novel randomized feature selection algorithms. Int. J. Found. Comput. Sci..

[19] Kursa MB, Rudnicki WR (2010). Feature selection with the Boruta package. J. Stat. Softw..

[20] van der Maaten L, Hinton G (2008). Visualizing data using t-SNE. J. Mach. Learn. Res..

[21] McCullagh P (2019). Generalized Linear Models.

[22] James G, Witten D, Hastie T, Tibshirani R (2013). An Introduction to Statistical Learning.

[23] Chen, T. & Guestrin, C. Xgboost: A scalable tree boosting system. In *Proceedings of the 22nd ACM SIGKDD International Conference on Knowledge Discovery and Data Mining* 785–794 (2016).

[24] Ripley, B., Venables, W. & Ripley, M. B. Package ‘nnet’. *R package version* **7**, 700 (2016).

[25] Meyer D, Wien F (2001). Support vector machines. R News.

[26] Günther F, Fritsch S (2010). Neuralnet: Training of neural networks. R J..

[27] Agrawal, A., Viktor, H. L. & Paquet, E. SCUT: Multi-class imbalanced data classification using smote and cluster-based undersampling. In *2015 7th International Joint Conference on Knowledge Discovery, Knowledge Engineering and Knowledge Management (IC3k)*, Vol. **1** 226–234 (IEEE, 2015).

