# Supplementary material for: Randomized feature selection based semi-supervised latent Dirichlet allocation for microbiome analysis
Source: Sci Rep. 2024 Apr 17;14:8855. doi: 10.1038/s41598-024-59682-4 (PMC11024186; doi:10.1038/s41598-024-59682-4)
Supplement: Supplementary file 1 — Supplementary Information. [file 41598_2024_59682_MOESM1_ESM.pdf]

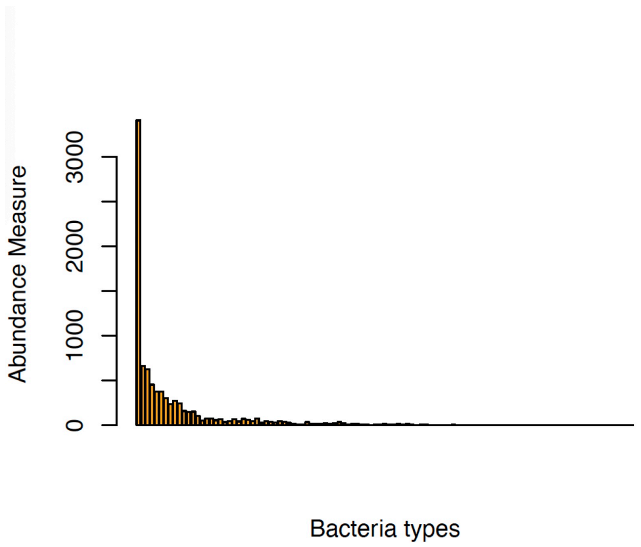

Figure S1. Empirical distributions of bacterial abundance measures.

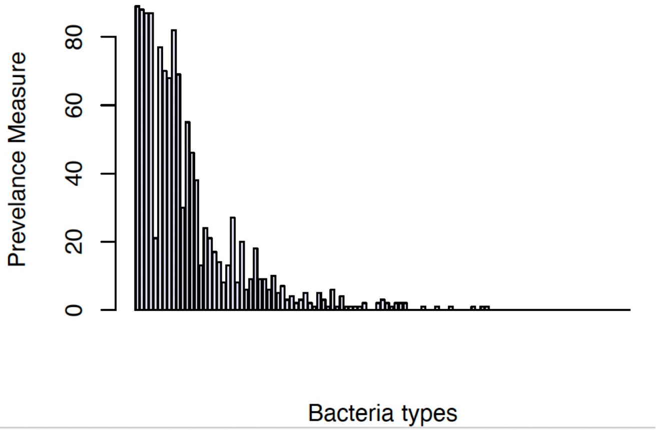

Figure S2. Empirical distributions of bacterial prevalence measures.

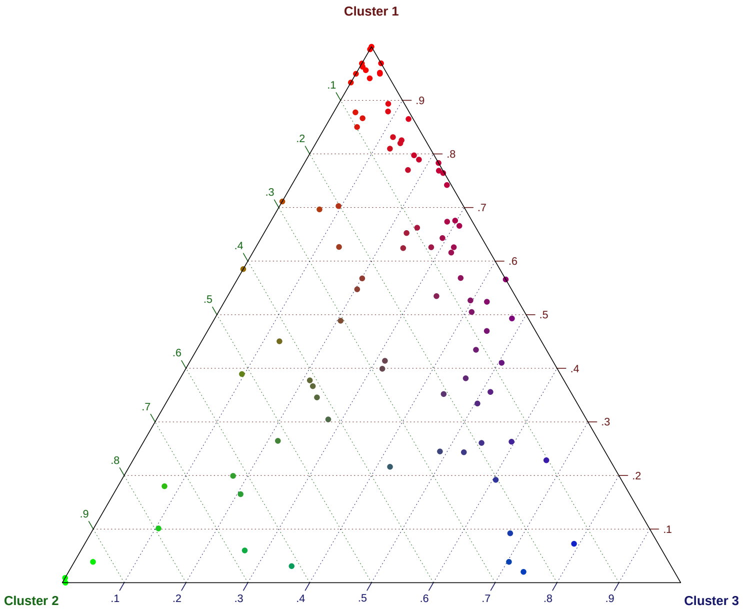

Figure S3. Clustering $M = 89$ subjects into $T = 3$ clusters using the LDA model

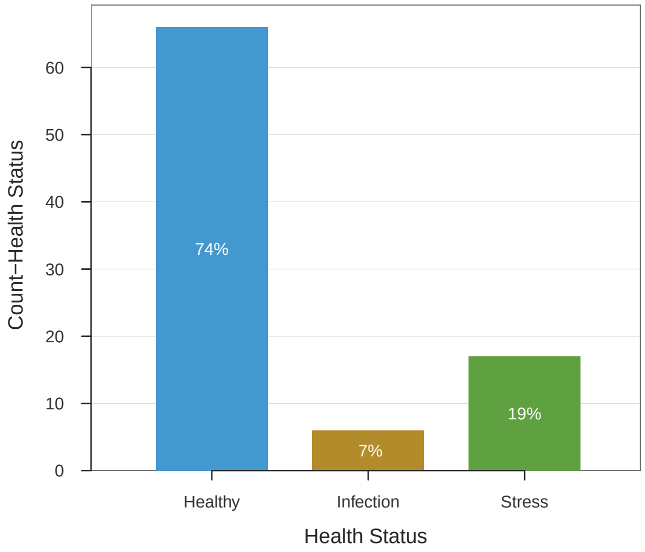

Figure S4. Barchart: Observed health status

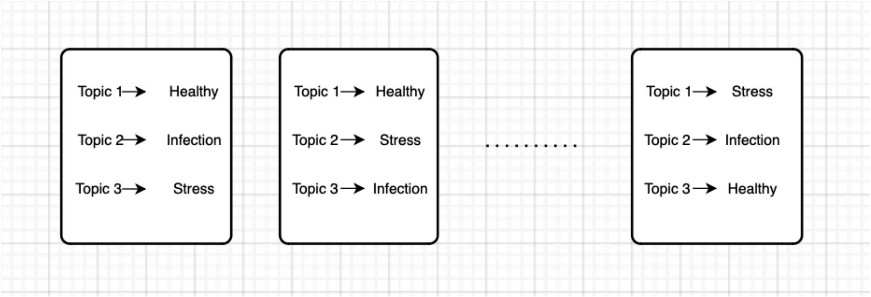

Figure S5. Three out of $T!$ scenarios for associating the topic labels and the observed health status

Notation	Description
M	Number of subjects.
B	Number of bacteria types.
$Y_{i,\ell}$	Overall read count of ℓth type of bacterium on the ith subject.
C_i	Health status of the ith subject.
T	Number health status levels/ Number of latent topics identified using LDA.
$p_{i,\ell}$	Proportion of ℓth bacterium level in subject i's microbiome.
$\mathbb{P}$	$M \times B$ proportion matrix.
ω	Detection threshold.
$I_{i,\ell}$	Indicates the presence or absence of ℓth bacterium level in subject i's microbiome based on detection threshold ω.
$\mathbb{I}$	$M \times B$ incidence matrix.
$\mathscr{A}$	Abundance of ℓth bacterium type.
$\mathscr{P}$	Prevalence of ℓth bacterium type.
K	top bacteria types obtained using the tau-path method.
B_0	$K+1$.
$Z_{i,\ell}$	Overall read count of ℓth top bacterium type on the ith subject.
b_ℓ	observed count of ℓth top bacterium type.
w_n	$B_0 \times 1$ vector indicates the bacteria type present in the nth position of $\mathscr{D}$.
$\pi'_{t,i}$	Estimated topic proportions on subject i for topic t.
π_i	Topic label corresponding to subject i.
w_t	Weights assigned to class level t.
λ	proportion threshold to identify within-group similarity.

Table S1. Description of Notations

w_1	w_2	w_3	Accuracy
0.5	0.15	0.35	0.4382
0.5	0.3	0.2	0.6067
0.6	0.15	0.25	0.7079
0.6	0.3	0.1	0.4944
0.7	0.15	0.15	0.6854
0.7	0.25	0.05	0.4382

Table S2. Results of the grid search used to select the candidate values for the weights

p	c	t_0	Accuracy
$n/2$	1	10^{-4}	0.6966
$n/2$	2	10^{-4}	0.6404
$n/4$	1	10^{-4}	0.7551
$n/4$	2	10^{-4}	0.6742

Table S3. Results of the grid search used to select the candidate values for the hyperparmetrs in RFSLDA
